# The Effect of Selenium Against Cadmium-Induced Nephrotoxicity in Rats: The Role of the TRPM2 Channel

**DOI:** 10.3390/toxics13020087

**Published:** 2025-01-24

**Authors:** Ömer Faruk Keleş, Mehmet Hafit Bayir, Hacı Ahmet Çiçek, Adem Ahlatcı, Kenan Yıldızhan

**Affiliations:** 1 Department of Pathology, Faculty of Veterinary Medicine, Van Yuzuncu Yil University, 65080 Van, Türkiye; haciahmet99@gmail.com; 2 Department of Histology, Faculty of Medicine, Van Yuzuncu Yil University, 65080 Van, Türkiye; mehmethafitbayir@yyu.edu.tr; 3 Vocational School of Health Services, Van Yuzuncu Yil University, 65080 Van, Türkiye; ademahlatci@yyu.edu.tr; 4 Department of Biophysics, Faculty of Medicine, Van Yuzuncu Yil University, 65080 Van, Türkiye; kenanyldzhan@gmail.com

**Keywords:** cadmium, selenium, TRPM2 channel, histopathology, immunohistochemically, rat

## Abstract

This study investigated the protective effect of selenium (Se) in a cadmium (Cd)-induced nephrotoxicity model in rats and the role of the TRPM2 channel in this mechanism. For this purpose, Cd (25 mg/kg orally), Se (0.5 mg/kg i.p.), and 2-aminoethoxydiphenyl borate (2-APB), a TRPM2 channel antagonist, (3 mg/kg i.p.) were administered to rats every day for 5 days. At the end of the study, kidney tissues were analysed using histological and biochemical methods. A histopathological examination revealed congestion, tubular degeneration, necrosis, and glomerular adhesion in the Cd group. However, these lesions were significantly reduced in the Cd + Se and Cd + 2-APB groups, while the Cd + Se + 2-APB group showed a histological appearance similar to the control group. Immunohistochemical analysis revealed that Caspase-3, Bax, and TRPM2 expression was higher in the Cd group, while these levels were lower in the Se and 2-APB treatment groups (*p* < 0.05). Among the groups that received Cd, urea, creatinine, TOS, TNF-α, and IL-1β levels were at the highest level in the Cd group, while TAS level was at the lowest level (*p* < 0.05). The Se and 2-APB treatment modulated these parameters; however, Se + 2-APB treatment reduced urea, creatinine, TOS, TNF-α, and IL-1β levels to the lowest level compared to the Cd groups and brought the TAS level closer to the control group (*p* < 0.05). These findings indicated that targeting TRPM2 channel inactivation together with the selenium treatment could alleviate Cd-induced nephrotoxicity.

## 1. Introduction

Technological development and industrialisation have brought increased exposure to many heavy metals through air, water, and food in daily life. Cadmium (Cd) is a very toxic heavy metal known in the world. The toxicological evaluation of heavy metals such as Cd is essential today because it is known that these agents cause severe toxicity in target organs after a certain period of exposure, even at low concentrations in predominantly nonsmoking women [1,2]. According to studies, oxidative stress and cadmium toxicity harm the antioxidant defence system, and lipid peroxidation is the main cause of cadmium toxicity [3,4].

The kidneys are the primary target organs adversely affected by heavy metal exposure, as they serve as sites for the accumulation of certain heavy metals [5]. It has been reported that Cd can cause many adverse effects on the kidneys, such as functional deterioration and diabetic nephropathy [6]. Oxidative stress and inflammation have been confirmed as the dominant mechanisms of Cd-induced nephrotoxicity [7,8]. It has also been determined that these mechanisms can lead to programmed cell death, such as apoptosis and autophagy. From a molecular perspective, it has been reported that the relationship between Cd exposure and nephrotoxicity affects DNA repair components, the production of reactive oxygen species (ROS) and the induction of apoptosis [9,10].

Selenium (Se) is an essential trace element found as a selenocysteine residue in the active site of selenoproteins and plays a role in the functions of various enzymes and proteins such as glutathione peroxidase (GPx), phospholipid–hydroperoxide glutathione peroxidase (PHGP), selenoprotein P, and thioredoxin reductases [11,12]. Most selenoproteins have anti-ROS properties; therefore, selenium is an essential element in cell survival through its antioxidant effect. The beneficial effects of Se treatment on Cd toxicity have been reported [13,14].

The melastatin-related transient receptor potential (TRPM) channel family plays crucial functions in the physiological processes of living creatures. TRPM2 is a protein expressed in many tissues. It involves many physiological processes, such as Ca^2+^ signalling in nerve cells, immune cells, and pain and insulin secretion from β-cells in the pancreas [15,16,17]. Various studies have addressed the effect of TRMP2 on extracellular Ca^2+^ signalling, which plays a central role in ROS production [18,19]. Studies on the pathophysiological importance of TRPM channels suggest that TRPM2 channels and their dependent mechanisms may be new potential therapeutic targets for immunity and various diseases [20,21]. Studies on the effects of the TRPM2 channel, known to be expressed in kidney tissue, on kidney pathophysiology are still insufficient. The 2-aminoethoxydiphenyl borate (2-APB), as an antagonist, is widely used as a pharmacological tool in studies on TRPM2 channel activation. In this study, 2-APB was used to investigate the role of the TRPM2 channel in the damage mechanism.

In this study, for the first time in the literature, TRPM2 channel inhibition and the protective effect of Se on Cd-induced kidney damage were investigated histopathologically, immunohistochemically, and biochemically. The results provide a multifaceted approach for therapeutics targeting Cd-induced kidney damage, revealing an approach that can target both Se for treatment and the TRPM2 channel mediator in the damage mechanism.

## 2. Materials and Methods

### 2.1. Antibodies and Chemicals

The Cd (Cadmium chloride, Cat#655198), 2-APB (Cat#D9754), and Se (Sodium selenite, Cat#214485) were supplied by commercial companies (Sigma-Aldrich Co., St. Louis, MO, USA). All other chemical components have very high purity grades. TRPM2 (Bioss Inc., Laval, QC, Canada, bs-2888R, dilution: 1/200), caspase 3 (Santa Cruz Biotechnology, Santa Cruz, CA, USA, sc-7272, dilution: 1/100), and Bax (Santa Cruz Biotechnology, sc-20067, dilution: 1/100) were all bought commercially. TAS (Reed Biotech, Wuhan, China, RBC0032), TOS (Reed Biotech, RBC0031), IL-1β (SinoGeneClon Biotech, Suzhou, China, Cat_SG-20260), and TNF-α (SinoGeneClon Biotech, Suzhou, China, Cat_SG-20127) levels were measured using commercially available ELISA kits following the kit procedures.

### 2.2. Ethics Statement and Groups of Experiments

For the study, 40 Albino Wistar male rats, 2–3 months old and weighing 200–300 g, were used. Ethics committee approval for the study was obtained from Van Yuzuncu Yil University Experimental Animals Local Ethics Committee (approval protocol number: 2024/09-04, date 26 September 2024). The rats were randomly divided into 5 groups, n = 8. The rats were housed in plastic cages with 12 h light and dark photoperiods at 24 °C. Standard feed and drinking water were given to the animals ad libitum throughout the experiment. This commercial standard rat food (Bayramoğlu Yem ve Flour Sanayi Ticaret A.Ş., Turkey) is widely used in the form of pellets prepared to provide sufficient nutrition for rats, detailed content is available in our previous study [22]. The effective dose and duration of Cd used were applied by considering the effective dose and duration previously used [23,24]. The effective dose and duration of Se was administered intraperitoneally (i.p.) at 0.5 mg/kg daily for 5 days, taking into account the study conducted by Yıldızhan et al. [25]. For the effective dose of 2-APB, the effective dose amounts and duration used by Thapak et al. were adjusted according to our study and applied [26]. Our study consisted of 5 groups, as stated below (n = 8).

***Control group:*** Rats in this group were given standard pellet feed daily for 5 days.

***Cd group:*** Rats in this group were given 25 mg/kg Cd via gavage for 5 days.

***Cd + Se group:*** In this group, Cd was given via gavage at 25 mg/kg for 5 days. The Se was administered i.p. every day for 5 days at 0.5 mg/kg.

***Cd + 2-APB group:*** In this group, Cd will be given i.p. at a 25 mg/kg dose for 5 days. 2-APB was given i.p. at a dose of 3 mg/kg every day for 5 days.

***Cd + Se + 2-APB group:*** In this group, Cd was given gavage at a 25 mg/kg dose for 5 days. 2-APB was given i.p. at a dose of 3 mg/kg every day for 5 days. The Se was also administered i.p. at 0.5 mg/kg daily for 5 days.

The study was terminated on the 5th day. The rats were deeply sedated using ketamine (50 mg/kg) and xylazine (20 mg/kg) anaesthesia in the laboratory of the same centre, and tissue and serum samples were collected. The serum samples were obtained by centrifuging 4 mL of blood samples in a dry biochemistry tube for 10 min at 3500/rpm and were stored at −80 °C until the study day. For biochemical analyses, some of the samples were stored at −80 °C. Some of the kidneys were placed in a 10% formaldehyde solution for histopathological and immunohistochemical examinations.

### 2.3. Histopathological Analysis

The kidney tissues were fixed in 10% formaldehyde, and routine procedures were followed. Then, 4 µm sections were taken from the tissues embedded in paraffin blocks and stained with hematoxylin and eosin (H&E). The sections were evaluated histopathologically under a light microscope (Olympus BX53, Tokyo, Japan).

### 2.4. Immunohistochemical Analysis for TRPM2, Bax, and Cas-3 Expressions

Next, sections that were 4 mm thick taken from paraffin blocks were incubated in a 3% hydrogen peroxide (H_2_O_2_) solution for 10 min to prevent endogenous peroxidase activity. Following routine procedures to prevent antigen masking in the nucleus and nonspecific binding, a primary antibody was added to the samples and incubated overnight at +4 °C in a humidified chamber. After rinsing with PBS, Biotinylated Goat Anti-Polyvalent (Anti-polyvalent HRP Kit; Termofisher Scientific, Waltham, MA, USA, PHL659660) and a Streptavidin–peroxidase conjugate were added to the samples and incubated for 10 min. After applying diaminobenzidine, which is used as a chromogen, the samples were stained with Mayer’s hematoxylin and evaluated under a light microscope (Olympus BX53, Japan). Immunohistochemical findings were evaluated subjectively according to the intensity of the staining in the tissue as negative (−), mild (+), moderate (++), and intense (+++) [27].

### 2.5. Biochemical Evaluation

Serum urea and creatinine were measured spectrophotometrically, and levels were measured by a turbidimetric method in a biochemical autoanalyser (Abbott Architect c16000, Chicago, IL, USA). TAS, TOS, IL-1β, and TNF-α levels in kidney tissues were measured using commercially available ELISA kits following the kit procedures. Protein amounts were calculated by comparing the absorbance values obtained with the standard curve. The previous procedures for protein amounts are given in detail in our earlier studies [28,29].

### 2.6. Statistical Analysis

The mean ± the standard error of the mean (SEM) was used to express all of the data. The Shapiro–Wilk test was used to assess the data’s normality, and the Levene test was used to verify that the variances of the independent groups were homogeneous. At the conclusion of the trial, every biochemical indicator displayed a normal distribution. Following the One-Way ANOVA analysis, the post hoc (Tukey HSD) test was conducted to ascertain which groups independently caused the difference because the data were normally distributed. Five percent was agreed upon as the statistical significance criterion. For statistical calculations, IBM SPSS Statistics for Windows 21.0 (IBM Corp., Armonk, NY, USA) was utilised.

## 3. Results

### 3.1. Histopathological Findings

A normal kidney histological structure was seen in the control group. Significant coagulation necrosis and degeneration in the proximal tubules, vascular congestion, and adhesion in the Bowman capsule were the most noticeable morphological alterations in the kidneys of the rats in the Cd group.

**Figure 1:**
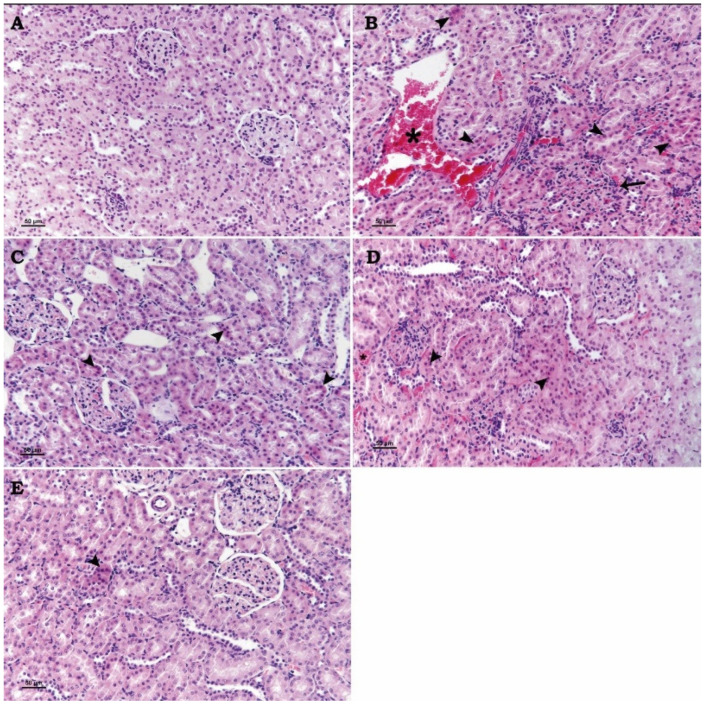
Light microscopic images of kidney tissue stained with hematoxylin and eosin (H&E). Group (**A**) (Control): The histological structure of the kidney appears normal. (**B**) (Cd Group): Coagulation necrosis and degenerations in the renal tubular epithelium (arrowheads), vascular congestion (*), and adhesion in the Bowman capsule (arrow). (**C**) (Cd + Se Group): Coagulation necrosis and degenerations in the renal tubular epithelium (arrowheads). (**D**) (Cd + 2-APB Group): Coagulation necrosis and degenerations in the renal tubular epithelium (arrowheads) and vascular congestion (*). (**E**) (Cd + Se + 2-APB Group): Coagulation necrosis and degenerations in the renal tubular epithelium (arrowhead). (Bar: 50 µm).

The kidneys of the Cd + Se and Cd + 2-APB groups were milder regarding these morphological changes than the Cd group rats. In addition, these morphological changes in the kidneys of the Cd + Se + 2-APB group rats were significantly reduced compared to the kidneys of the Cd + Se and Cd + 2-APB groups (Figure 1).

### 3.2. Immunohistochemical Findings

**Figure 2:**
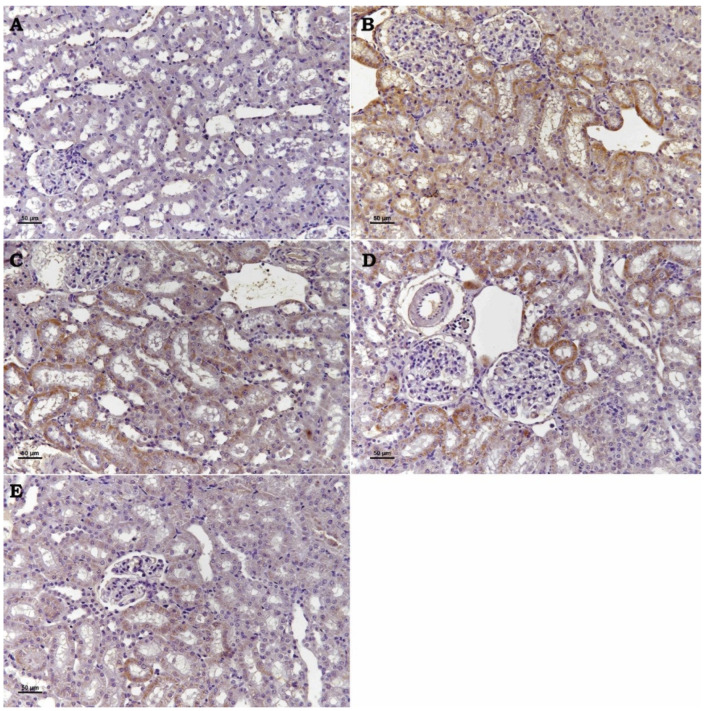
Se and 2-APB’s impact on TRPM2 channel expression in kidney injury caused by Cd. TRPM2 channel expression was detected by immunohistochemical staining. (**A**) Control, (**B**) Cd, (**C**) Cd + Se, (**D**) Cd + 2-APB, and (**E**) Cd + Se + 2-APB. (Bar: 50 µm).

**Figure 3:**
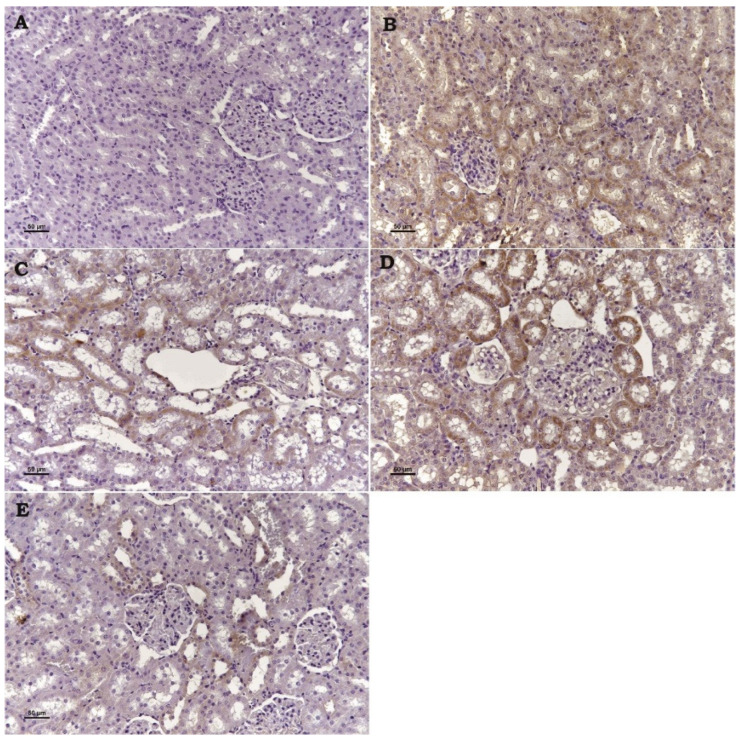
Se and 2-APB’s impact on Bax expression in kidney injury caused by Cd. Bax expression was detected by immunohistochemical staining. (**A**) Control, (**B**) Cd, (**C**): Cd + Se, (**D**) Cd + 2-APB, and (**E**) Cd + Se + 2-APB. (Bar: 50 µm).

**Figure 4:**
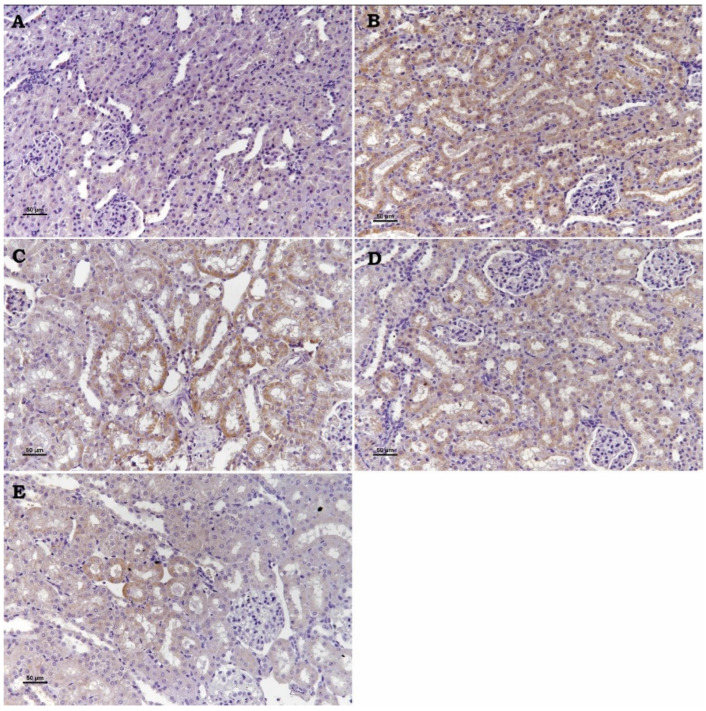
Se and 2-APB’s impact on Cas-3 expression in kidney injury caused by Cd. Cas-3 expression was detected by immunohistochemical staining. (**A**) Control, (**B**) Cd, (**C**) Cd + Se, (**D**) Cd + 2-APB, and (**E**) Cd + Se + 2-APB. (Bar: 50 µm).

TRPM2 channel (Figure 2), Bax (Figure 3), and Cas-3 (Figure 4) Expressions: These three markers applied to the kidney tissues of all rats produced negative reactions in the control group, a high positive expression in the Cd group, a moderate positive expression in the Cad + Se and Cad+ APB groups, and a low positive reactions in the Cad + Se + APB group (Table 1).

### 3.3. Changes in Renal Function Markers in Cd-Induced Kidney Damage

**Figure 5:**
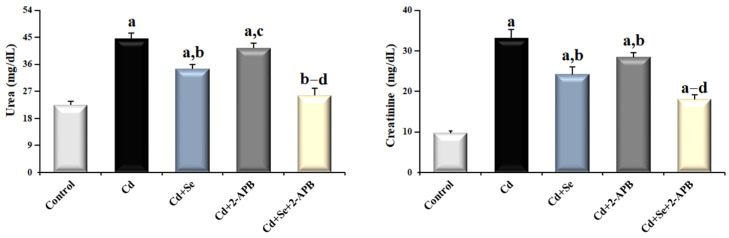
The effect of Se and TRPM2 channel inactivation on urea and creatinine in Cd-induced kidney damage. (Mean ± SD., and n = 8). (^a^ *p* < 0.05 vs. control group, ^b^ *p* < 0.05 vs. Cd group, ^c^ *p* < 0.05 vs. Cd + Se group, ^d^ *p* < 0.05 vs. Cd + 2-APB group).

In Figure 5, when serum samples taken from rats were examined, Cd administration significantly increased urea and creatinine levels, which are kidney function tests, compared to all other groups (*p* < 0.05). The urea and creatinine levels of the Cd + Se group were lower compared to the Cd and Cd + 2-APB groups (*p* < 0.05). The urea and creatinine levels of the Cd + Se + 2-APB group were the lowest compared to the other Cd-administered group (*p* < 0.05).

### 3.4. Changes in TAS and TOS Levels in Cd-Induced Kidney Damage

**Figure 6:**
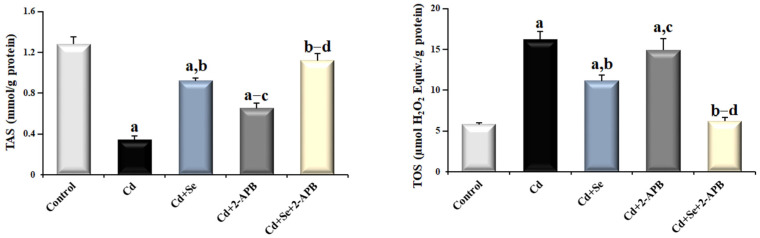
The effect of Se and TRPM2 channel inactivation on TAS and TOS levels in Cd-induced kidney damage. (Mean ± SD., and n = 8). (^a^ *p* < 0.05 vs. control group, ^b^ *p* < 0.05 vs. Cd group, ^c^ *p* < 0.05 vs. Cd + Se group, ^d^ *p* < 0.05 vs. Cd + 2-APB group).

When TAS levels were measured in rats’ kidney tissue (Figure 6), the Cd group’s TAS level was the lowest compared to the other groups (*p* < 0.05). The TAS level of the Cd + Se group was significantly higher compared to the Cd and Cd + 2-APB groups *(p* < 0.05). While there was no significant difference between the TAS levels of the Cd + Se + 2-APB group and the control group (*p* > 0.05), the TAS level of the Cd + Se + 2-APB group was the highest compared to the other Cd-administered groups (*p* < 0.05).

Compared to the other groups, the Cd group had the greatest TOS level (*p* < 0.05). When the Se and 2-APB treatment groups were examined with Cd application, Se treatment decreased the TOS level compared to 2-APB (*p* < 0.05). In addition, in the combined use of Se and 2-APB (Cd + Se + 2-APB), the lowest TOS level was observed among the groups using Cd (*p* < 0.05).

### 3.5. Changes in Pro-Inflammatory Markers in Cd-Induced Kidney Damage

**Figure 7:**
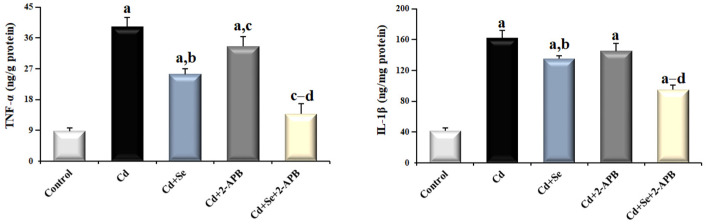
The effect of Se and TRPM2 channel inactivation on IL-1β and TNF-α markers in Cd-induced kidney damage. (Mean ± SD., and n = 8). (^a^ *p* < 0.05 vs. control group, ^b^ *p* < 0.05 vs. Cd group, ^c^ *p* < 0.05 vs. Cd + Se group, ^d^ *p* < 0.05 vs. Cd + 2-APB group).

Figure 7 shows tissue samples from rats. TNF-α and IL-1β levels were considerably higher after Cd treatment than in the other groups (*p* < 0.05). Compared to the Cd and Cd + 2-APB groups, the TNF-α level of the Cd + Se group was lower (*p* < 0.05). The Cd + Se + 2-APB group had the lowest levels of TNF-α and IL-1β when compared to the other Cd-administered group (*p* < 0.05).

## 4. Discussion

Cadmium is a heavy metal found in many of our environments (air, water, soil, and sediment) [30]. Cadmium accumulates largely in the kidneys and causes tubular diseases. Exposure to cadmium through various routes causes organ dysfunction as a result of cell death [23]. Selenium (Se), an active component of selenoproteins, is a mineral with antioxidant function and a wide range of protective effects on health [11,12]. It has been reported in many studies that the TRPM2 channel is a Ca^2+^ permeable channel closely related to oxidative stress [31,32]. In addition, activation of TRPM2 by H_2_O_2_ is thought to be involved in various cell deaths. [33]. In this study, the relationship and protective effect of selenium with 2-APB, a TRPM2 channel inhibitor, in Cd-induced kidney injury were investigated histopathologically, immunohistochemically, and biochemically.

When the studies on renal toxicity and damage induced by different agents, methods and heavy metals (ischemia, glycerol, CdCl_2_, HgCl_2_, experimental rhabdomyolysis, cisplatin, and cyclosporine A) are examined, the changes reported in the damage groups are different from the control groups, including congestion, degenerative necrotic changes in glomeruli and tubules, inflammatory cells and dilatation in peritubular areas, epithelial deformation, vacuolations, fibrosis, hyalinosis, and thickening of tubule epithelium [34,35,36,37,38,39]. In our study, degenerative necrotic changes, coagulation necrosis, and congestion were observed in glomeruli and tubules in common with the findings of other investigators. In addition to these, adhesion in the bowman capsule was noted. In our findings, vacuolations, fibrosis, and hyalinosis were not observed. Considering the similarity of the findings to a great extent, our application for renal toxicity is considered to be successful.

In a study investigating the chemoprotective effect of selenium nanoparticles (Se-NPs) in Cd-induced kidney injury [36], Se-NPs were shown to be positively effective in CdCl_2_-induced nephrotoxicity by preventing apoptosis and altering cell protective pathways. Cd exposure has been reported to cause congestion in the glomeruli, marked enlargement of Bowman’s capsule and excessive damage to the glomerular epithelium. In our study, congestion, tubular degeneration necrosis, and glomerular adhesion were observed in Cd-exposed groups. These lesions were decreased in the Cd + Se group compared to the Cd group. In this context, our findings are consistent with the researchers in terms of the protective-healing effect of selenium. In addition, Cd + 2-APB group rats showed fewer lesions in the kidneys compared to the Cd group. This finding revealed the role of TRPM2 channels in cell apoptosis and showed that 2-APB, a TRPM2 channel inhibitor, was successful. Indeed, in the Cd + Se + 2-APB group, in which cadmium, selenium, and 2-APB were administered together, lesions were significantly reduced compared to Cd + Se and Cd + 2-APB groups. Our biochemistry and immunohistochemistry results support these findings.

In a study conducted to evaluate whether selenium nanoparticles (SeNPs) can provide protection against the glycerol-induced acute kidney injury (AKI) model, it was stated that selenium nanoparticles can be used against AKI [35]. The researchers reported that tubular dilatation, vacuolation, necrosis, and debris accumulation in the tubular lumen were observed in the glycerol-treated group of rats, while these lesions improved in the group given SeNPs. In addition, they stated that SeNPs significantly inhibited the increased TNF-α, IL-1β, urea, and creatinine levels and decreased their expression compared to glycerol-treated rats. In our study, according to the findings of the researchers, the lesions in the kidneys of Cd + Se and Cd + 2-APB group rats were decreased compared to Cd group rats. In addition, the kidneys of Cd + Se + 2-APB group rats recovered at a level close to the control group. These findings support our opinion that 2-APB administration may further increase the effect of selenium. The changes in TNF-α, IL-1β, urea and creatinine values in our study are also consistent with our histopathology findings and the reports of the researchers.

In a study investigating the role of TRMP2 in ischemia-induced renal injury, it was reported that renal tubular damage was similar in all ischemia duration groups and TRMP2 inhibitor groups [32]. These authors argue that TRMP2 channels do not play an important role in acute kidney injury. In our study, the kidney damage in Cd + 2-APB group rats was significantly less than the kidney damage in Cd group rats. In addition, degenerative necrotic changes in the kidneys of Cd + Se group rats were improved compared to the Cd group. However, degenerative-necrotic changes in the kidneys of Cd + Se + 2-APB group rats were significantly better than the other treatment groups. Our findings are not compatible with the findings of the researchers. As a matter of fact, in our study, TRMP2 immunohistochemistry staining findings clearly showed that TRMP2 channels were inhibited and the damage decreased in proportion to the inhibition in this context and increased the effect of selenium. These findings indicate the negative role of TRMP2 channels in renal damage and that the effect of selenium on the kidney may be more beneficial by the inhibition of these channels.

In a study examining the effects of N-acetyl cysteine (NAC) on TRPM2 expression in malathion-induced renal toxicity, it was reported that TRPM2 immunoreactivity significantly increased in malathion and malathion + NAC groups compared to the control group, whereas TRPM2 immunoreactivity decreased in malathion + pralidoxime + atropine groups and malathion + pralidoxime + atropine + NAC groups compared to the toxicity groups [40]. In our study, TRPM2 immunoreactivity gave the highest reaction in the Cd group, while the reaction decreased in the Cd + 2-APB group. In addition, TRPM2 immunoreactivity in the Cd + Se + 2-APB group was close to the control group. Our findings were found to be consistent with the information provided by the above researchers regarding the effect of TRMP2 channels in the kidneys.

In a study in which the effect of 8-bromo-cyclic ADP-ribose (8-Br-cADPR; acADPR antagonist), a TRPM2 channel inhibitor, on a renal ischemia–reperfusion injury, was investigated histopathologically, immunohistochemically, and biochemically; it was reported that 8-Br-cADPR administration reduced the ischemia–reperfusion injury in accordance with the dose increase. The investigators observed congestion, connective tissue proliferation, inflammatory cell infiltration in the peritubular area, necrosis, dilatation, and epithelial degeneration in the tubules in the histopathological sections of the ischemia–reperfusion injury in the group of rat kidneys. They reported that such findings were attenuated in the groups in which 8-Br-cADPR was administered at low doses and improved in the groups in which it was administered at high doses, close to the control group. In addition, in the immunohistochemical evaluation of TRMP2 ion channels and Caspase-3, which is one of the cell apoptosis markers, they reported that the damage and immunohistochemical positivity created in proportion to histopathological examination decreased in the 8-Br-cADPR-treated groups by the increase in dose. In the biochemistry results of these researchers, it was suggested that 8-Br-cADPR decreased TNF-α and IL-1β levels by the dose increase compared to damaged kidney tissues. They also stated that urea creatinine levels decreased in 8-Br-cADPR-treated groups compared to I/R-treated groups [34]. In our study, the histopathologic findings (congestion, tubular degeneration, necrosis, and adhesion) observed in the kidneys of Cd group rats regressed in Cd + Se and Cd + 2-APB groups compared to the Cd group. In the kidneys of Cd + Se + 2-APB group rats, it regressed more than the other groups and the findings were almost absent. In our study, TRPM2 and Caspase-3 immunohistochemistry results showed that the damage and immune positivity decreased in the Cd + Se and Cd + 2-APB groups compared to the Cd group, and further decreased in the Cd + Se + 2-APB group rats compared to the Cd + Se and Cd + 2-APB groups. In our biochemistry results, TNF-α, IL-1β, urea, and creatinine levels decreased in the Cd + Se and Cd + 2-APB groups compared to the Cd group, and this decrease was significantly increased in the Cd + Se + 2-APB group. In this context, our findings are consistent with the findings of the above researchers. In addition, the Bax immunohistochemistry evaluation in our study was compatible with our TRPM2 and Caspase-3 immunohistochemistry results. In addition, our findings in TAS and TOS parameters support the positive effect of 2-APB administration. When the findings of the above researchers and our findings are evaluated, the researchers support our views that 2-APB, a TRPM2 channel inhibitor, has a positive effect on nephrotoxicity and selenium increases the protective treatment efficacy.

In a study aiming to reveal the possible protective effect of dapagliflozin (DAPA) against cyclosporine A-induced acute renal failure, it was reported that DAPA administration significantly reduced CsA-induced histopathological findings (parenchymal inflammation, hyaline pattern formation, vacuolization, and lysis of renal tubular cells) and increased TAS and TOS parameters [41]. In our study, lesions in Cd group kidneys were reduced in Cd + Se, Cd + 2-APB, and Cd + Se + 2-APB groups. When the TAS and TOS parameters of our study were compared with the above researchers, they were found to be compatible in terms of the TOS parameter, but not in terms of the TAS parameter. As a matter of fact, contrary to the researchers, the TAS level in the damage group was the lowest in our study compared to all other groups. Our TOS values were the highest in the damage group and decreased in the other groups by the researchers.

## 5. Conclusions

In this study, the therapeutic effect of Se on Cd-induced nephrotoxicity (25 mg/kg Cd daily by gavage for five days) was investigated in rats. In addition, the role of the TRPM2 cation channel in this damage mechanism in Cd-induced nephrotoxicity was investigated in the study using 2-APB, a TRPM2 channel antagonist. As a result of renal function tests, oxidative stress markers, pro-inflammation levels, and histopathological and immunohistochemical examinations, it was determined that the Se treatment and the TRPM2 channel inactivation reduced the damage against Cd-induced nephrotoxicity. In a histopathological evaluation, significant coagulation necrosis and degeneration in proximal tubules and vascular congestion and adhesion in Bowman’s capsule were observed in the kidneys of rats in the Cd group. It was determined that the Se treatment and 2-APB use alleviated the histopathological damage observed due to Cd. In Cd-induced nephrotoxicity, it was observed that the Se treatment and 2-APB use effectively restored renal function by reducing creatinine and urea levels, which improved renal health and modulated increased oxidative stress and inflammatory levels in Cd-induced kidneys. Immunohistochemical studies showed that TRPM2, Bax, and Cas-3 expression levels increased after Cd application and decreased with Se treatment and 2-APB use compared to the Cd group. The findings of this study showed that the Se treatment and TRPM2 channel inactivation reduced Cd-induced nephrotoxicity. To better understand the therapeutic properties of Se in Cd-induced nephrotoxicity and the role of the TRPM2 channel in this mechanism, different biochemical markers and parameters related to TRPM2 activation should be investigated at the molecular level and in more detail.

## Figures and Tables

**Table 1:** The intensity of the TRPM2, Bax, and Cas-3 immunoreactivity in the rat kidney tissues.

	Groups				
	Control	CdCI_2_	CdCI_2_ + Se	CdCI_2_ + 2-APB	CdCI_2_ + Se + 2-APB
TRPM2	−	+++	++	++	+
Bax	−	+++	+	++	+
Casp-3	−	+++	++	+	+

Negative (−), mild (+), moderate (++), and intense (+++).

## Data Availability

The data that support the findings of this study are available from the corresponding author upon reasonable request. Data can be obtained from the authors upon request.
